# Single-Center Evaluation of Treatment Success Using Two Different Protocols for MRI–Guided Transurethral Ultrasound Ablation of Localized Prostate Cancer

**DOI:** 10.3389/fonc.2021.782546

**Published:** 2021-10-27

**Authors:** Gencay Hatiboglu, Valentin Popeneciu, David Bonekamp, Mathieu Burtnyk, Robert Staruch, Florian Distler, Jan Philipp Radtke, Johann Motsch, Heinz Peter Schlemmer, Sascha Pahernik, Joanne Nyarangi-Dix

**Affiliations:** ^1^ Department of Urology, University of Heidelberg, Heidelberg, Germany; ^2^ German Cancer Research Center Deutsches Krebsforschungszentrum (DKFZ), Heidelberg, Germany; ^3^ Profound Medical Inc., Mississauga, ON, Canada; ^4^ Department of Anaesthesiology, University of Heidelberg, Heidelberg, Germany

**Keywords:** TULSA, success, outcome, phase 1 clinical studies, pivotal

## Abstract

**Objectives:**

To assess differences in 24-month oncologic and functional outcomes in men with low to intermediate-risk prostate cancer treated with MRI-guided transurethral ultrasound ablation (TULSA) using intentionally conservative versus intensified treatment parameters.

**Patients and Methods:**

Patients from a single center involved in two multicenter trials were included in this analysis. This included 14 of 30 patients with Gleason 3 + 3 from a Phase I study using intentionally conservative treatment parameters, and 15 of 115 patients with Gleason ≤ 3 + 4 from a pivotal study using intensified parameters. Follow-up data compared across these cohorts included 12-month biopsy and MRI for all patients, and 24-month PSA, micturition and quality of life (IIEF, IPSS, IPSS-QOL). The prognostic value of baseline parameters and PSA kinetics on 12-month histological recurrence was evaluated by logistic regression.

**Results:**

12-month biopsy revealed clinically significant residual disease in 4 (29%) and 2 (14%) patients from the Phase I and pivotal studies, respectively. PSA nadir was 0.7 ng/ml for Phase I and 0.5 ng/ml for pivotal study patients. Patient age at diagnosis, use of MRI fusion/systematic prostate biopsy, number of obtained cores at initial biopsy, PSA course, and PSA nadir were identified as prognostic factors for treatment success. All but one patient from each cohort maintained erection firmness sufficient for penetration. No cases of pad use were reported at 24 months. There were no Grade 4 or higher adverse events, and no late toxicity related to the procedure.

**Conclusion:**

Two-year follow-up demonstrated the efficacy of TULSA for the treatment of localized prostate cancer, and the durability of PSA and functional outcomes. Intensifying treatment parameters in the pivotal trial had no impact on safety or functional outcomes through 24 months, while reducing the recurrence rate for clinically significant disease. Careful patient selection by MRI fusion/systematic prostate biopsy and adequate follow-up through routine 12-month biopsy are recommended.

## Introduction

Men with early stage low – or intermediate risk prostate cancer face different therapy options for treatment. Although, standard therapy like active surveillance, radical prostatectomy or radiotherapy provide excellent oncological and functional results in experienced centres (1), some patients seek for alternative treatment options with less procedure-related side effects. MRI-guided transurethral ultrasound ablation (TULSA) of prostate tissue is an emerging technology for thermal coagulation of diseased prostate tissue that has the advantages of intraoperative MRI-based treatment planning and automated treatment control based on real-time MRI thermometry (2). A Phase I trial in patients with localized prostate cancer (PCa) demonstrated clinical feasibility and safety (3). As feasibility rather than oncological effect was the main purpose of that evaluation, treatment parameters were intentionally conservative by sparing 3 mm of prostatic tissue within the capsule, leaving 10% of the prostate volume untreated, resulting in residual disease in up to 55% of patients at 12-month prostate biopsy (3). For the subsequent pivotal study of treatment efficacy, treatment parameters were intensified to achieve complete whole-gland ablation (4).

As a center that enrolled and treated patients in both multicenter trials, we previously presented a single-center comparison of the initial 6-month safety outcomes between patients in the Phase I and pivotal trials (5). By comparing patients treated by the same physicians in both trials, the surgeon’s impact on treatment outcome could be excluded. We demonstrated that there were no significant differences in sexual function, continence, or other adverse events associated with intensifying treatment parameters to achieve ablation to the prostate capsule. However, the short follow-up time in that report did not allow for comparison of oncological outcomes, and did not fully capture the recovery of functional outcomes. Here, we provide the first comparison of oncologic outcomes in patients treated in the Phase I and pivotal trials with 24-month follow-up including 12-month biopsy, and evaluate prognostic factors for treatment success.

## Patients and Methods

The prospective, multicenter, single-arm Phase I trial (NCT01686958) recruited thirty male patients ≥65 years with biopsy-proven PCa (clinical stage T1c–T2a, N0, M0), PSA ≤10 ng/ml, and Gleason score 3 + 3 between March 2013 and March 2014. Recruitment of men with Gleason score 3 + 4 was allowed at one of the three sites in the Phase I study, but not at our institution. The multicenter TACT pivotal trial (NCT02766543) enrolled 115 men ≥45 years (clinical stage T1c-T2b, N0, M0), PSA ≤15 ng/ml), and Gleason score on biopsy of 3 + 3 or 3 + 4. All men in the pivotal trial were treated between September 2016 and February 2018.

For this single-center analysis, only patients treated at the University of Heidelberg (Germany) were included: 14 patients treated in Phase I and 15 patients treated in the pivotal trial.

Both trials were approved by the institutional ethics board and written informed consent was obtained from all study participants. All patients at our institution were treated by the same surgeons.

### TULSA Procedure

Men in both studies were treated with the TULSA-PRO system (Profound Medical Inc., Mississauga, Canada) (3, 5–8). The treatment was performed under general anesthesia in a 3T MRI unit. A robotic positioning system (PS) was used to control the linear and rotational motion of the 10-element transurethral ultrasound applicator (UA) within the prostate under MRI-guidance, with each element emitting high-intensity, directional ultrasound. A 3-mm safety margin was preserved towards the apical sphincter in both studies. Treatment plans were defined according to high-resolution MRI images which were acquired continuously during treatment to provide real-time MRI thermometry feedback of the ablation (3). For treatment planning, the urologist and the radiologist marked the target volume by defining the outer boundaries of the prostate.

### Treatment Plans

**Figure 1:**
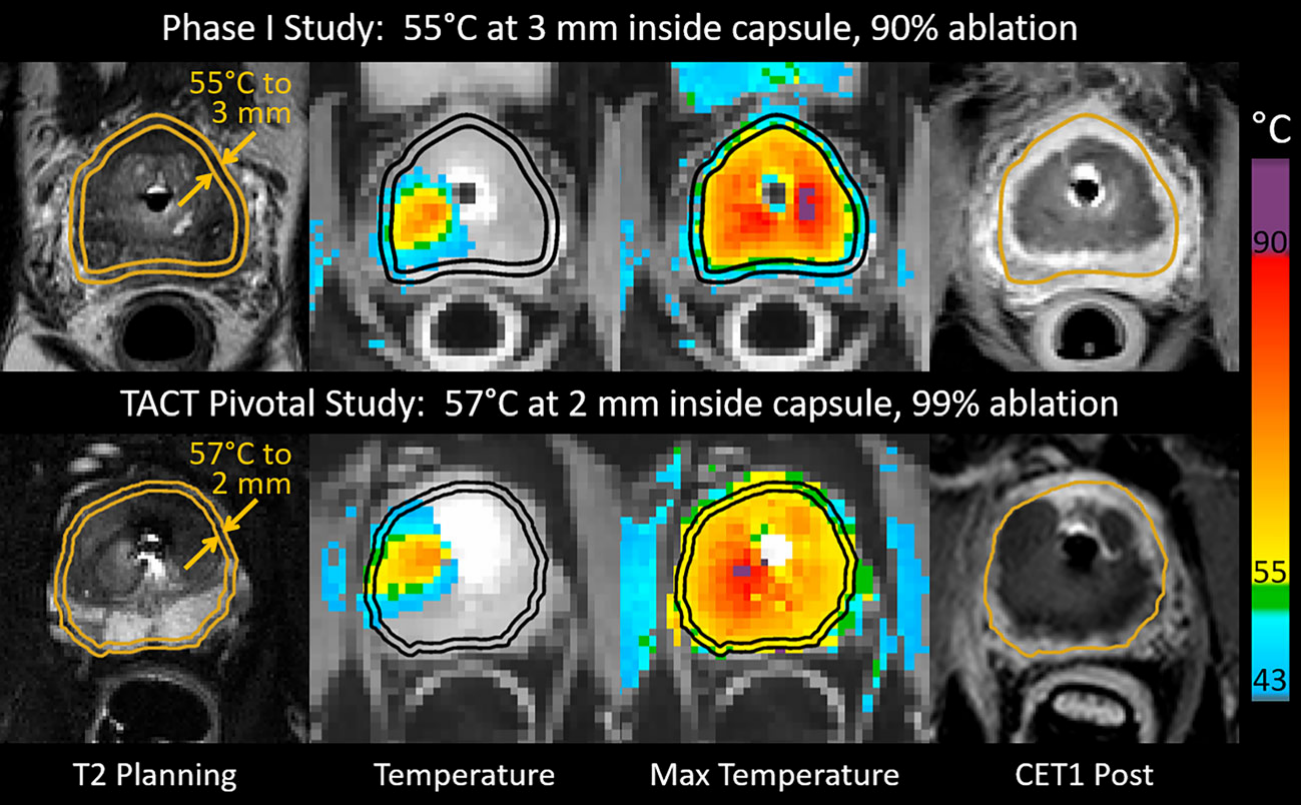
Intraoperative images from patients who underwent MRI-guided transurethral ultrasound ablation (TULSA) using conservative treatment parameters in the Phase I study (top), and intensified parameters designed to achieve whole-gland ablation in the pivotal study (bottom). Transverse images at one location in the midgland for each patient depict interoperative treatment planning on T2-weighted images, real-time MRI temperature images used to control treatment, maximum temperature projections used to assess ablation coverage during treatment, and post-treatment contrast-enhanced T1-weighted images (CET1) demonstrating greater ablation extent in the example from the pivotal study (bottom).

Treatment differences between the Phase I and pivotal trials have been described previously (5). The main differences were a reduction in the safety margin of expected tissue preservation inside the prostatic capsule from 3 mm (Phase I) to less than 1 mm (pivotal trial), which was achieved by an increase of the treatment control temperature from 55°C (Phase I) to 57°C (pivotal), and a reduction of the minimum rotational speed of the UA from 8 (Phase I) to 4 (pivotal) degrees per minute. These changes to treatment parameters were expected to deliver immediate cell kill ≥ 55°C within 1 mm of the prostate capsule and to increase the ablation coverage from 90% to 99% of the targeted prostate volume (**Figure 1**).

### Follow-Up Schedule

All patients underwent physical examination, ultrasound, and blood chemistry, and PSA at the baseline visit. A multiparametric MRI of the prostate was done at baseline in all patients and rated according to the Prostate Imaging Reporting and Data System (PI-RADS) (9). The first follow-up was after 2 weeks to remove the suprapubic catheter and evaluate micturition. Additional follow-up visits took place at 1, 3, 6, 12, 18, and 24 months. At each visit, patients were evaluated for adverse events, PSA, and blood chemistry, and a physical examination was performed. Validated questionnaires were administered at baseline and at all follow-up visits, these were: the International Prostate Symptom Score (IPSS), IPSS quality of life (IPSS-QoL), and the International Index for Erectile Function (IIEF-15). Patient´s continence was evaluated by interview and pad usage. At the 12-month visit, a follow-up multiparametric MRI of the prostate and a prostatic biopsy (≥12 core) was performed per protocol to evaluate oncological treatment outcome. In addition, non-perfused prostatic volume (contrast-enhanced T1w) and ablation coverage (measured by MR thermometry) was calculated for all patients after treatment and compared between groups (10) (**Figure 1**).

### Statistical Analysis

Above mentioned variables were evaluated by descriptive statistics. Median, mean, standard deviation and interquartile range were calculated for every variable. Difference testing between baseline parameters for the two treatment groups (Phase I and pivotal) was performed using the chi-square test for categorical variables and Mann-Whitney-U test for continuous variables. Difference testing was also performed for oncologic and functional outcomes at 12 to 14-month follow-up. Univariate analysis was done by logistic regression to assess the impact of selected covariates on the risk of clinically significant cancer (defined as high volume Gleason=3 + 3 or Gleason > 3 + 3) at 12 months, for the pooled cohort of patients treated in both studies and with completed follow-up. A p value <0.05 was considered statistically significant. All statistical analyses were carried out using IBM SPSS software package version 26.

## Results

The main differences in baseline characteristics between the Phase I and pivotal cohorts were the inclusion of younger men with higher-risk Gleason 3 + 4 disease in the pivotal study (**Table 1**). As expected, treatment-day MR thermometry indicated that a larger proportion of the prostate reached an ablative thermal dose in the pivotal study participants (median 98%, IQR 97-99%) than in the Phase I study (90%, IQR 88-92%). The proportion of non-enhancing tissue on immediate post-treatment contrast-enhanced MRI was not significantly different (pivotal study: 56%, IQR 52-71% vs. phase I: 51%, IQR 44-58%, p=0.57).

**Table 1:** Clinical baseline and pathological characteristics between the Phase I and pivotal trial patients treated at our institution.

		Phase 1 N = 14	Pivotal N = 15	P-value
Age, median (IQR)		71.0 (69.2-73.0)	67.0 (64.9-71.9)	0.10
Prostate Volume, median (IQR)		41.0 (33.8-65.7)	44.5 (33.4-54.5)	0.76
PSA, median (IQR)		6.6 (4.0-8.1)	6.6 (4.5-7.3)	0.79
Gleason Score				**0.01**
Gleason 3 + 3		14	9	
Gleason 3 + 4		0	6	
IIEF, median (IQR)		11.5 (3.8-26.5)	22.5 (5.0-28.8)	0.21
IPSS, median (IQR)		8.5 (5.0-15.5)	10.0 (5.5-14.8)	0.74
IPSS quality of life, median (IQR)		2.5 (1.8-4.0)	3.0 (2.0-3.8)	0.91
Number of pads, median (IQR)		0 (0-0)	0 (0-0)	1.00
Testosterone, median (IQR)		4.2 (3.7-5.4)	3.8 (2.3-5.5)	0.36
Mode of initial biopsy				0.74
12 core TRUS		11	11	
MRI fusion/systematic biopsy		3	4	
Number of obtained biopsy cores median (IQR)		25.5 (16.0-28.0)	27.0 (16.0-30.0)	0.67
Number of positive biopsy cores median (IQR)		3.0 (2.0-4.3)	4.0 (1.0-6.0)	0.95

Groups were compared by chi-square tests for categorical variables and Mann-Whitney-U tests for continuous variables. A p value < 0.05 was considered statistically significant.

Statistical significant values are displayed in bold.

### Mid-Term Oncologic Outcomes

Mid-term oncological follow-up data for both studies are presented in **Table 2**. Biopsy findings at 12 months were available for all but one man in each cohort, who withdrew from the study with low PSA. PSA measurements at 24 months were available for 12 of 14 in Phase I (one withdrawal and one who underwent salvage treatment before 24 months), and 9 of 15 in the pivotal study (four withdrawals with low PSA, two salvage). Post-TULSA PSA nadir in Phase I was 0.7 ng/ml (IQR 0.2-0.8) at a median of 3 months (IQR 1.0-6.0); PSA nadir in the pivotal trial was 0.5 ng/ml (IQR 0.2-1.2) reached within 3 months (IQR 3.0-4.5). Follow-up biopsies at 12 months detected clinically significant cancer (high volume Gleason=3+3 or any Gleason >3+3) in four patients (29%) of the Phase I study and two patients (14%) in the pivotal trial. Clinically insignificant findings (small volume Gleason 3 + 3) were detected in one additional patient in Phase I, and three additional patients in the pivotal trial.

**Table 2:** Oncological follow-up: PSA values at baseline and follow-up, PSA nadir, time to PSA nadir and results of 12 months prostate biopsy for phase 1 and pivotal trial patients.

		Phase 1 N = 14	Pivotal N = 15	P-value
PSA, median (IQR)				
Baseline		6.6 (4.0-8.1)	6.6 (4.5-7.3)	0.79
1 month		0.9 (0.5-2.0)	0.8 (0.4-1.5)	0.78
3 months		0.9 (0.3-1.7)	0.5 (0.2-1.1)	0.13
6 months		0.7 (0.4-1.3)	0.5 (0.3-1.4)	0.60
12 months		0.9 (0.6-1.7)	1.0 (0.7-1.5)	0.82
24 months		0.9 (0.4-2.5)	0.9 (0.4-2.2)	0.86
PSA Nadir at 12 months		0.7 (0.2-0.8)	0.5 (0.2-1.2)	0.42
Time to PSA nadir		3.0 (1.0-6.0)	3.0 (3.0-4.5)	0.45
Any Recurrence at 12 months biopsy (%)		5/13 (38.5%)	5/14 (35.7%)	0.88
Gleason Score (recurrence)				0.10
Gleason 3 + 3		2	4	
Gleason 3 + 4		0	1	
Gleason 4 + 3		3	0	
Clinically significant		4 (28.6%)	2 (14.3%)	0.2

Groups were compared by Mann-Whitney-U test. A p value < 0.05 was considered statistically significant.

**Table 3** compares all patients with cancer recurrence to those with negative 12-month biopsy. At time of diagnosis, men who eventually had recurrence were significantly younger (p=0.02), had lower cancer risk classification (p=0.03), had fewer biopsy cores sampled at initial diagnosis (p=0.02), and were less likely to have had a MRI fusion/systematic initial biopsy (p=0.03). During follow-up, men who had recurrence on 12-month biopsy had higher PSA at nadir (p=0.01), higher PSA values at 3 to 12-month visits, and had higher PIRADS scores at 12-month multiparametric MRI (p=0.01).

**Table 3:** Comparison of patients with and without cancer recurrence.

		No recurrence N = 17	Recurrence N = 10	P-value
Age, median (IQR)		71.7 (68.8-73.9)	68.2 (54.8-70.2)	**0.016**
Prostate Volume, median (IQR)		44.0 (35.4-51.9)	43.1 (31.8-58.0)	0.980
Initial PSA, median (IQR)		6.7 (5.0-7.8)	6.6 (4.7-7.9)	0.880
PSA 3 months, median (IQR)		0.5 (0.2-1.3)	1.2 (0.7-2.7)	**0.031**
PSA 6 months, median (IQR)		0.5 (0.3-0.9)	1.2 (0.8-1.7)	**0.017**
PSA 12 months, median (IQR)		0.8 (0.5-1.2)	1.4 (1.0-2.5)	**0.007**
PSA nadir 12 months Median (IQR)		0.5 (0.2-0.6)	1.0 (0.6-1.3)	**0.010**
Gleason Score				0.097
Gleason 3 + 3		12	10	
Gleason 3 + 4		4	0	
Risk classification				**0.033**
Low risk		11	10	
Intermediate risk		6	0	
Initial Biopsy				**0.029**
TRUS		2	5	
MRI fusion/systematic		15	5	
Initial MRI				0.184
Yes		17	9	
No		0	1	
IIEF, median (IQR)		11.0 (3.5-28.0)	22.5 (10.5-29.0)	0.172
IPSS, median (IQR)		8.0 (4.5-14.5)	10.0 (7.5-14.3)	0.465
Quality of life, median (IQR)		3.0 (2.0-4.0)	2.5 (1.8-4.0)	0.938
Testosterone, median (IQR)		4.0 (2.7-6.0)	4.8 (4.0-5.3)	0.547
Percentage Non-Perfused Volume (%)		54.0 (47.5-61.0)	53.5 (37.3-57.3)	0.407
No of biopsy cores (initial biopsy)		28.0 (20.5-30.5)	17.5 (12.3-26.5)	**0.023**
No of pos. cores (initial biopsy)		3.0 (1.5-5.0)	3.5 (1.8-4.5)	0.839
PIRADS Score (initial biopsy)		4 (3-4)	4 (4-5)	0.337
No of biopsy cores Follow-Up		12.0 (12.0-15.0)	12.5 (12.0-14.8)	0.847
Any Lesion follow-up MRI				0.883
Yes		8	5	
no		9	5	
PIRADS score at 12 months		3 (3-4)	4 (4-5)	**0.008**
Treatment protocol				0.883
Phase 1 Study		8	5	
Pivotal Trial		9	5	

Groups were compared by Mann-Whitney-U test for continuous variables and chi-square for categorial variables. A p value < 0.05 was considered statistically significant.

Statistical significant values are displayed in bold.

Univariate analysis of predictive factors for any cancer recurrence at 12-month biopsy was performed including all patients from both studies (**Table 3**). Hereby, patient age (OR:0.71; p=0.03), PSA at 12 months (OR:4.01; p=0.04), PSA nadir (OR:16.52; p=0.02), mode of initial biopsy (OR:7.5; p=0.04) and number of biopsy cores sampled at initial biopsy (OR:0.87; p=0.03) had statistically significant impact on oncological outcome. Intensifying treatment parameters had no statistically significant impact on cancer recurrence. A separate univariate evaluation focusing on patients with clinically significant tumor recurrence revealed PSA at 12 months (OR: 4.3; p=0.04) and PSA nadir (OR: 13.2; p=0.03) as predictive factors.

Patients with clinically significant tumor recurrence were referred for salvage treatment. In the Phase I cohort, two patients underwent salvage radiation therapy and two patients salvage prostatectomy. In the pivotal trial, the two patients with clinically significant tumor recurrence both underwent salvage prostatectomy. Patients with clinically insignificant tumor recurrence are under surveillance with regular PSA controls and scheduled re-biopsies. One patient of our Phase I cohort discontinued follow-up to pursue active surveillance before the one-year visit, and four patients in our pivotal cohort withdrew from the study to pursue active surveillance before the two-year visit.

### Functional Outcomes and Safety

Functional outcomes for patients in the Phase I and pivotal studies are listed in **Table 4**. Catheterization durations were longer in pivotal study versus Phase I patients: median 20.0 (IQR 10-42) days vs median 14.5 (IQR 13-25) days, respectively (p=0.18).

**Table 4:** Functional follow-up: Comparison of catheter indwelling time, IPSS, quality of life and pad usage for evaluation of micturition and IIEF questionnaire data for erectile function at baseline and during follow-up for phase 1 and pivotal trial patients.

	Phase 1 N = 14	Pivotal N = 15	P-value
Catheter indwelling time, median (IQR)	14.5 (13.0-25.0)	20.0 (10.3-42.0)	0.18
IPSS, median (IQR)			
Baseline	8.5 (5.0-15.5)	10.0 (5.5-14.8)	0.74
1 month	15.5 (11.0-21.0)	14.5 (11.5-18.5)	0.84
3 months	5.0 (3.0-10.0)	7.5 (3.5-10.0)	0.38
6 months	5.0 (3.0-7.0)	7.0 (2.0-9.0)	0.50
12 months	6.0 (3.5-6.5)	4.5 (1.8-10.5)	0.77
24 months	8.5 (6.0-9.0)	3.0 (2.5-9.5)	0.18
Quality of life, median (IQR)			
Baseline	1.5 (0.8-3.0)	1.0 (1.0-3.0)	0.86
1 month	3.0 (1.0-3.0)	4.0 (2.0-6.0)	0.12
3 months	1.0 (0.0-1.0)	2.0 (1.0-3.0)	**0.01**
6 months	0.5 (0.0-1.0)	1.0 (0.0-2.0)	0.06
12 months	1.0 (0.0-1.0)	1.0 (0.0-2.3)	0.50
24 months	0.5 (0.0-1.0)	0.0 (0.0-1.5)	0.78
Number of pads (IQR; range)			
Baseline	0 (0.0; 0.0)	0 (0.0; 0.0)	1.00
1 month	0 (0.0; 0.0)	0 (0.0; 0.2)	**0.04**
3 months	0 (0.0; 0.0)	0 (0.0; 0.1)	0.33
6 months	0 (0.0; 0.0)	0 (0.0; 0.0)	1.00
12 months	0 (0.0; 0.0)	0 (0.0; 0.0)	1.00
24 months	0 (0.0; 0.0)	0 (0.0; 0.0)	1.00
IIEF, median (IQR)			
Baseline	11.5 (3.8-26.5)	25.0 (8.0-29.0)	0.20
1 month	3.0 (1.0-9.3)	4.5 (1.8-9.3)	0.58
3 months	11.5 (4.8-16.5)	14.0 (1.0-29.0)	0.54
6 months	11.0 (5.3-20.0)	14.0 (2.0-25.0)	0.98
12 months	19.0 (8.0-25.0)	14.5 (8.8-25.0)	1.00
24 months	17.5 (3.8-24.3)	7.0 (1.5-25.5)	0.86
IIEF Q2 erection sufficient for penetration			
Baseline	2.5 (0.8-5.0)	4.0 (0.0-5.0)	0.69
1 month	0.0 (0.0-1.5)	0.0 (0.0-1.8)	0.57
3 months	2.0 (0.0-3.3)	2.5 (0.0-4.8)	0.37
6 months	2.5 (1.0-4.3)	2.5 (0.0-4.8)	0.95
12 months	4.0 (2.5-4.0)	3.5 (1.8-5.0)	0.94
24 months	3.0 (0.3-4.8)	1.0 (0.0-4.5)	0.58

Groups were compared by Mann-Whitney-U test. A p value < 0.05 was considered statistically significant.

Statistical significant values are displayed in bold.

After an initial worsening of urinary symptoms at 1 month in both groups, IPSS recovery was seen in both groups at 3 and 6 months. At 12 to 24 months, the IPSS score returned to baseline in both cohorts, with improvement beyond baseline noted in pivotal patients (median 56% improvement from baseline to 24 months). IPSS quality of life was improved compared to baseline starting at 3 months in Phase I and 6 months in the pivotal study, with most men from both trials reporting that they were “pleased” or “delighted” with their urinary condition at 24 months. At one month, four pivotal study patients reported the use of pads related to urine leakage, but by the 6-month visit all men in both cohorts were pad-free and remained so at 2 years.

Between baseline and 24 months, only one patient from each study lost the ability to achieve erections sufficient for penetration (IIEF question 2 ≥ 2). IIEF-15 erectile function domain scores showed recovery by 3 months with wide variability in both groups.

Perioperative adverse events to 6 months have been reported previously (5). No late adverse events related to the procedure were noted beyond 6 months.

## Discussion

We compare two different treatment protocols for TULSA with regards to functional and oncologic outcomes at 2 years for the subgroup of men treated at our institution. By this means, we also assess the impact of intensified treatment parameters and identify prognostic factors for treatment success. Whole-gland prostate ablation delivered using MRI-guided TULSA led to lower rates of clinically significant tumor recurrence compared with wide safety margins. The intensified parameters had no impact on clinical safety and minimal impact on functional outcomes at 24 months.

In the Phase I trial, safety and treatment precision were the primary study outcomes, not oncological efficacy. A wide safety margin of 3 mm inside the prostate capsule was intentionally spared regardless of cancer location, leaving a rim of viable prostate tissue (10). In the subsequent pivotal study designed to assess treatment efficacy, treatment parameters were intensified to remove the safety margin and increase treatment temperature and exposure time (by reducing minimal rotation speed). This reduced safety margins to the prostatic capsule and increased ablation coverage from 90% to 99% of the gland (5). For the subgroups of men treated at our center, the rate of clinically significant disease on the 12-month biopsy was reduced from 29% to 14% by intensifying treatment parameters, similar to the reduction from 31% to 15% seen in the full cohorts (2, 4).

Despite more aggressive treatment plans, there were no material differences in urinary function between the two subgroups from 6 to 24 months. Four of our pivotal study patients reported pad use due to urgency and mild incontinence at 1 month, but recovered to pad-free continence by six months, as did all of our patients in the Phase I study. IPSS and IPSS quality of life scores showed an initial increase in both subgroups, followed by an improvement to better than baseline levels in the pivotal study. Bladder outlet obstruction as a typical side effect of other thermoablative treatments (11, 12) did not occur in any of our patients.

The incidental, ameliorating effect of TULSA treatment on lower urinary tract symptoms (LUTS) has recently been described for a subgroup of the overall population of Phase I patients who entered the study with cancer and concomitant LUTS (13). However, a comparison between the Phase I and TACT cohorts is described here for the first time. While micturition symptoms reported through IPSS scores recovered to baseline by 2 years in the Phase I subgroup, improvements relative to baseline were seen in the pivotal study subgroup at the 24-month visit. We attribute the enhanced urinary symptom relief to the higher treatment temperatures and exposure times applied in the pivotal study, in line with previous in-vitro studies demonstrating that increased thermal dose improved ablation effect for hyperplastic prostate tissue (14, 15).

Baseline erection firmness sufficient for penetration (IIEF question 2 ≥2) was maintained for all but one patient from each subgroup. However, IIEF-15 erectile function domain scores trended towards improved recovery in the Phase I subgroup. The reduced ablation safety margins of the pivotal study were expected to result in increased heating to within 1mm of the neurovascular bundles adjacent to the prostatic capsule, compared to the wide 3mm subcapsular margin applied in the Phase I study which may have resulted in better preservation of erectile function. While the differences were not statistically significant in this small sample size, they suggest that the intensified parameters per the pivotal trial whole-gland ablation protocol could be considered non-nerve-sparing, whereas a safety margin of 3 mm appears to be suitable for regions of the gland where nerve-sparing is intended and oncologically acceptable.

In our evaluation, the PSA course and especially PSA nadir were significant predictors for tumor recurrence. As previously demonstrated, PSA nadir is a prognostic indicator for disease-free survival in patients undergoing HIFU treatment (16, 17) or radiotherapy (18). Definition of a threshold for biochemical recurrence post-TULSA awaits long-term outcomes and evaluation of larger cohorts. In these cohorts with ablation of at least 90% of the prostate, 75% of those with nadir ≤ 0.6 ng/ml were disease-free, while 75% with nadir above 0.6 ng/ml had histological recurrence. Of four men with histological recurrence after nadir ≤ 0.6 ng/ml, two with only one core of residual Gleason Grade 1 disease had PSA remain within nadir + 0.5 ng/ml thereafter. The other two each had 3/12 positive cores and presence of residual Gleason Grade 2 disease, and had PSA increase to nadir + 0.7 and nadir + 1.2 ng/ml by one year, eventually exceeding nadir + 2.0 ng/ml. Routine follow-up biopsy at 12 months is therefore recommended to ensure timely detection of residual disease. For patients with tumor recurrence, standard salvage treatments like radical prostatectomy (19) or radiotherapy (20) are viable options.

Another factor that influenced tumor recurrence at 12 months was the use of stereotactic or MRI-guided biopsy at baseline. The accuracy of MRI guided stereotactic biopsy has been described before and showed significantly better diagnostic accuracy (21, 22). The combination of MRI fusion biopsy with systematic biopsy has shown even better accuracy in detecting clinically significant prostate cancer (23) or the index tumor (24) than 12-core systematic biopsy alone. In the Phase I trial, more of our patients had a previous MRI-guided or stereotactic biopsy than in other centers (3), while the pivotal trial mandated preoperative multiparametric prostate MRI for all patients. We assume that our center’s lower recurrence rate in Phase I compared to other centers is related to the initial mode of biopsy. The increased diagnostic certainty accessible with MRI-guided or stereotactic biopsy is essential for appropriate patient counseling and decision-making, and was correlated with decreased tumor recurrence at 12 months.

Limitations of this evaluation include the short follow-up of 24 months and the small cohorts. In addition, the treatment of predominantly intermediate risk cancers in the pivotal trial must be taken into consideration while only men with low-risk cancer were treated at our center in Phase I. Furthermore, two-year follow-up was lacking for 6 of 15 men in the pivotal study: four who declined additional follow-up having low PSA, and two who had undergone salvage treatment. Although clinical safety and functional outcome can be evaluated, the data on oncological outcomes need further evaluation with a longer follow-up.

## Conclusion

In patients at one institution involved in two different multicenter studies, intensified treatment parameters for whole-gland prostate ablation using MRI-guided TULSA led to lower rates of clinically significant tumor recurrence while having no impact on clinical safety or 24-month functional outcomes. PSA course and especially PSA nadir, as well as baseline use of MRI fusion biopsy technique predicted histopathological evidence of residual disease. Close follow-up through PSA monitoring with 12-month MRI and biopsy is recommended for early detection of disease recurrence.

## Data Availability Statement

The raw data supporting the conclusions of this article will be made available by the authors, without undue reservation.

## Ethics Statement

The studies involving human participants were reviewed and approved by Ethikkommission Heidelberg, Universität Heidelberg. The patients/participants provided their written informed consent to participate in this study.

## Author Contributions

GH, project development, data collection, data analysis, manuscript writing and editing. VP, data collection and data analysis. DB, data collection, data analysis, manuscript writing and editing. MB and RS, project development, data analysis, manuscript writing and editing. FD and JR, data analysis, manuscript writing and editing. SP, project development, data collection, and data analysis. JM, data collection and supervision. HS, project development, supervision, and critical revision of manuscript. JN-D, data analysis, manuscript writing and editing, and critical revision of manuscript. All authors contributed to the article and approved the submitted version.

## Conflict of Interest

RS and MB are employees of Profound Medical and receive a salary and stock options.

The remaining authors declare that the research was conducted in the absence of any commercial or financial relationships that could be construed as a potential conflict of interest.

## Publisher’s Note

All claims expressed in this article are solely those of the authors and do not necessarily represent those of their affiliated organizations, or those of the publisher, the editors and the reviewers. Any product that may be evaluated in this article, or claim that may be made by its manufacturer, is not guaranteed or endorsed by the publisher.

## Abbreviations

IIEF, international index for erectile function; IPSS, international prostate symptom score; IQR, interquartile range; MRI, magnetic resonance imaging; PCa, prostate cancer; PS, positioning system; QoL, quality of life; TACT, TULSA-PRO Ablation Clinical Trial; TULSA, transurethral ultrasound ablation; UA, ultrasound applicator.
